# Total trade, cereals trade and undernourishment: new empirical evidence for developing countries

**DOI:** 10.1007/s10290-022-00468-z

**Published:** 2022-05-09

**Authors:** Marta Marson, Donatella Saccone, Elena Vallino

**Affiliations:** 1 Department of Economics and Statistics “Cognetti de Martiis”, University of Turin, Lungo Dora Siena 100, 10153 Turin, Italy; 2 OEET-Turin Center on Emerging Economies, Collegio Carlo Alberto, Piazza V. Arbarello 8, 10122 Turin, Italy; 3 University of Gastronomic Sciences, Piazza Vittorio Emanuele II, 9, 12042 Pollenzo, Bra, CN Italy; 4 Department of Cultures, Politics and Society, University of Turin, Lungo Dora Siena 100, 10153 Turin, Italy

**Keywords:** Undernourishment, Food security, Trade openness, Cereals trade, F6, F13, Q1, Q18

## Abstract

While trade policies are considered strategic to shape national food systems and promote food security, the ultimate impact of trade openness on hunger is still highly debated. Using a sample of 81 developing over the period 2001–2016 and principally focusing on the prevalence of undernourishment, this study provides new empirical evidence. Firstly, it estimates the impact of total trade differentiating the effects that pass through changes in real per capita income—i.e. on the economic access to food—from the residual effects that it directly has on the other dimensions of food security. Subsequently, it concentrates on cereals trade, that usually is the most affected by trade restrictions and the most correlated to undernourishment. Finally, it explores the different effects of cereals trade in terms of imports and exports. Three main conclusions emerge: (a) trade openness contributes to lower the prevalence of undernourishment in developing countries and most of this effect is not income-mediated but, rather, passes through the impacts that it directly has on the other dimensions of food security; (b) such impacts are mostly driven by the trade openness of the cereals sector where (c) its import component turns out to play the main role.

## Introduction

With the launch of the Sustainable Development Goals (SDGs) in 2015, food security has been fully recognized as a global goal.^1^ The seventeen SDGs, defined in the 2030 Agenda for Sustainable Development, represent a “plan of action for people, planet and prosperity” signed by 193 UN Member States and including 169 associated targets to be achieved by 2030 (UN, 2015). In particular, Goal 2 “*Zero hunger*” calls for ending hunger and ensuring “access by all people to safe, nutritious and sufficient food all year round” (UN, 2015, Target 2.1). This specific target is mainly measured through the percentage of undernourished population (SDG Indicator 2.1.1, UN, 2019b)^2^ and is highly interconnected to the other SDGs through important synergies (Fader et al., 2018; UN, 2019a).

**Fig. 1:**
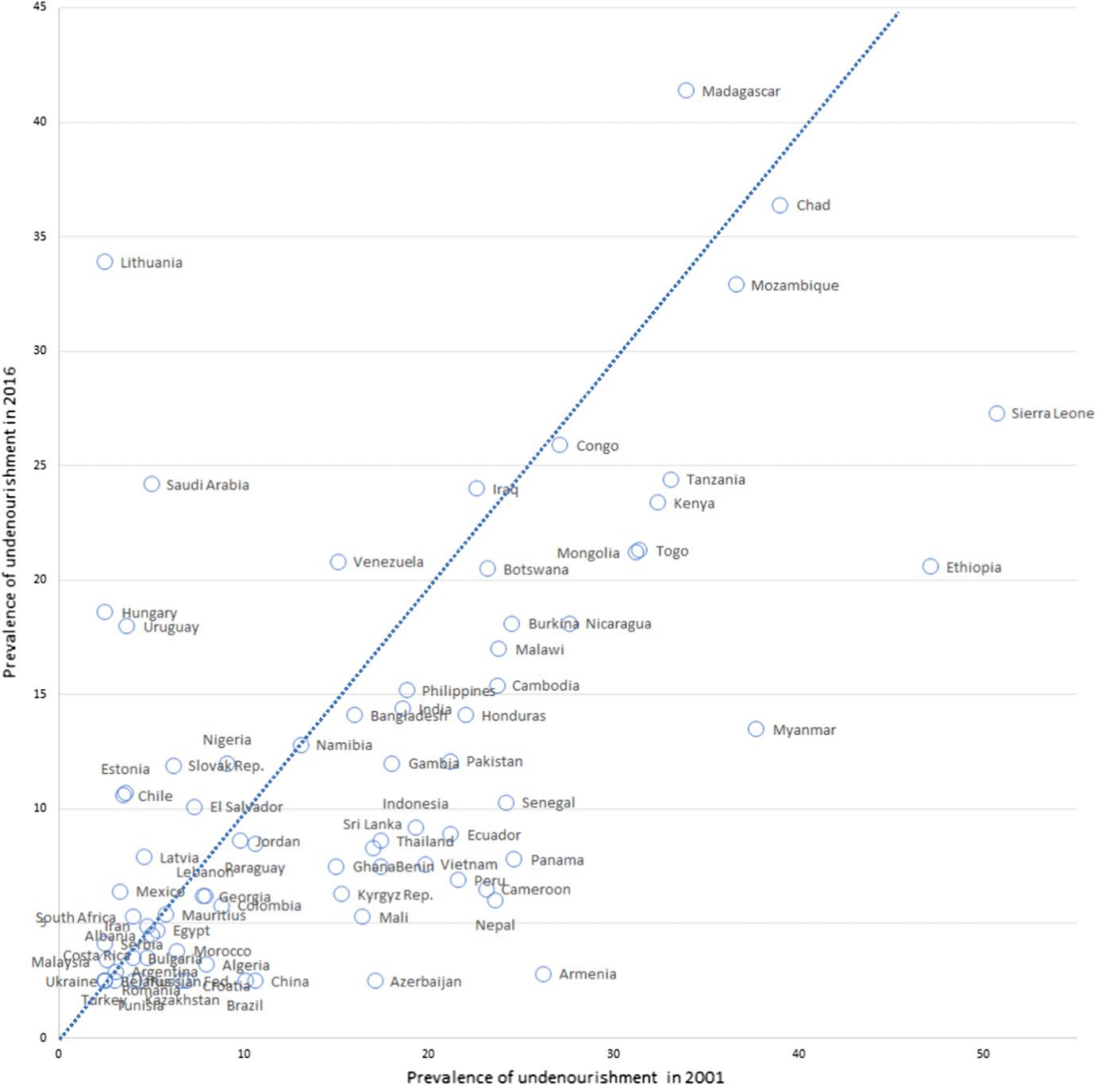
Prevalence of undernourishment (2001–2016). *Source*: Own elaborations on FAO data

Since the beginning of the new millennium remarkable improvements have been recorded in the prevalence of undernourishment, with most countries now presenting lower percentages than at the beginning of the 2000s (see also Fig. 1). However, in absolute terms, the number of undernourished people is increasing again and, currently, nearly 768 million people (corresponding to 9.9% of the world population) suffer from hunger globally. Moreover, the first FAO’s estimates suggested that the Covid-19 pandemic could have caused an additional 83–132 million people in the ranks of the undernourished (FAO et al., 2020). The outbreak is also causing important changes in global food supply chains and in the agricultural trade policies of many countries, which resorted to temporary export restrictions (FAO, 2020; Kerr, 2020; WTO, 2020). These figures pose challenges related to all dimensions of food security (food availability, food accessibility, food utilization, and stability: FAO et al., 2020) and to the resilience of food systems (Béné, 2020; Hansen et al., 2020).

This created further constraints to free trade along with the US-China trade war and the subsequent resurgence of protectionism (Fajgelbaum et al., 2020). While trade policies are considered strategic to shape national food systems and promote food security, the ultimate impact of trade openness on hunger is still highly debated. Moreover, the empirical evidence is relatively scarce and fragmented if compared to the large literature about the effects of trade on economic growth, poverty, inequality and other aspects of development (see Ravallion, 2018; Irwin, 2019; Lechthaler & Mileva, 2019; Ramirez-Rondàn et al., 2020). Most of the literature on food security and trade, in fact, is either qualitative (see, for example, FAO, 2015) or mainly focused on very specific aspects—such as agricultural prices (among others, Flachsbarth & Garrido, 2014; Headey, 2011)—or on single countries (Davalos et al., 2020; Dorosh & Rashid, 2013; Dorosh et al., 2009; Montalbano et al., 2018; Porteous, 2017).

Only recently two important cross-country studies have aimed to estimate the average impact that trade openness has on food security. On a panel of 151 developed and developing countries (1980–2007), Dithmer and Abdulai (2017) find that trade openness is positively related to both average dietary energy and diversity. Mary (2019) better delineates the scope of the analysis by only focusing on developing countries and distinguishing between the effects of food and non-food trade. Using a sample of 52 developing countries (1990–2013), he finds that food trade openness is positively related to undernourishment prevalence.

In general, while these studies represent important contributions to depict the impact of trade on food security at a macro level, their results do not fully converge and there is still large room for further investigation. Using a sample of 81 developing countries over the period 2001–2016 and principally focusing on the prevalence of undernourishment (SDG Indicator 2.1.1, UN, 2019b), our study aims at providing new empirical evidence and contributes to the debate in four ways. *First*, it starts by estimating the impact of total trade openness disentangling the effects that pass through changes in real per capita income—i.e. the effects on the economic access to food-from the residual effects that it directly has on the other dimensions of food security. *Second*, since these latter effects seem to prevail, the paper further deepens the focus by concentrating on food trade and, specifically, on cereals trade. The importance of focusing on cereals trade is based on the relevance of staple food in the overall calorie intake as well as in food trade (Brooks & Mattews, 2015; Traverso & Schiavo, 2020; Wright, 2012) and in related restrictions, as shown by the centrality of cereals in recent export bans (Porteous, 2017).

*Third*, the paper explores the different effects of cereals trade in terms of imports and exports. The two sides of trade can in fact have diverging effects on food security, as it will be discussed in Sect. 2. For example, based on economic theory, imports can impact on undernourishment through food inflows and changes in domestic food markets caused by higher competition and lower prices, while exports can give rise to food outflows and changes in income due to greater access to global markets and higher prices. This can also be reflected in a different effect of trade in net cereals-importing and exporting countries. To this purpose, after estimating the main models for the full sample, the analysis is separately carried out for the two groups of countries.^3^*Fourth*, from a methodological point of view, by taking inspiration from Romalis (2007), the analysis accounts for potential endogeneity by instrumenting trade openness with the trade openness of the rest of the world, after its validity is discussed and tested in Sect. 3.

The paper is structured as follows. Drawing from the existing micro and macro literature, Sect. 2 theoretically discusses the potential positive and negative effects that trade can have on the different dimensions of food security. Section 3 describes data and methodology. Section 4 presents the empirical results, while Sect. 5 concludes and points out the policy implications.

## The potential impacts of trade on food security

Before reviewing the literature on the relation between trade openness and food security, it is useful to mention the more general links that exist between trade and health. Owen and Wu (2007), by assessing a panel of 219 countries, find that higher trade openness is associated with positive health outcomes, among which less infant mortality and higher life expectancy, in particular in developing countries. Potential channels are knowledge spillovers and good economic policies, which are also associated to a higher openness. More recently, Novignon et al. (2018) find similar results on a set of 42 Sub-Saharan African countries (1995–2013), also unveiling a positive effect of trade openness on health financing. In the field of nutrition, there is an ongoing debate on the impact of globalization and trade openness on overnutrition and obesity. Many authors identify positive and significant association between openness and/or globalization and obesity, with stronger effect for developing countries (among others An et al., 2019; De Vogli et al., 2014; Giuntella et al., 2020), while others argue that the impact of trade openness on overnutrition in developing countries is overstated and the rise in obesity may be better explained by general modernization trends than trade itself (Fox et al., 2019; Mary & Stoler, 2021). The debate on the relation between trade openness and food security is therefore inserted in a more complex framework that concerns the wider domain of the connection between globalization and health.

There exists a large body of converging studies investigating the macroeconomic (Bellemare et al., 2013; Burchi & De Muro, 2016; Candelise et al., 2021; Dhahri & Omri, 2020; Drèze & Sen, 1989; Green & Kirkpatrick, 1982; Headey & Martin, 2016; Sen, 1981; Smith & Haddad, 2002; Soriano & Garrido, 2016) and microeconomic (Behrman & Deolalikar, 1987; Bouis & Haddad, 1992; Haddad et al., 1996; D’Souza & Jolliffe, 2010; Bellemare & Novak, 2017; Tiwari 2019; Brown et al., 2019) determinants of food security. A part of the scientific literature addresses food security at the system level, by focusing on the resilience of food systems, intended as “all the elements (environment, people, inputs, processes, infrastructures, institutions, etc.) and activities that relate to the production, processing, distribution, preparation consumption [and waste management] of food, and the output of these activities, including socio-economic and environmental outcomes” (Béné, 2020).

Within this framework, the results of the literature on the relation between trade openness and food security are relatively fragmented and the ultimate impact of the first on the latter is still highly debated (Dithmer & Abdulai, 2017; Mary, 2019). The lack of clear-cut conclusions derives from the complexity of the relation, where the outcome results from the combination of the effects of trade on diverse dimensions of food security. In order to build a general framework that systematizes the results of the extant literature and guides the interpretation of our empirical results, we discuss here the potential positive and negative impacts of trade on the main dimensions of food security (availability, access, utilization) and their stability. Such impacts are summarized in Table 1.

**Table 1:** Potential impacts of trade openness on food security (Authors elaboration)

Potential positive impacts		Potential negative impacts	
Better food availability and diversity through increased efficiency in domestic food production and higher food inflows	Burgess and Donaldson (2010), Dorosh and Rashid (2013), Baldos and Hertel (2015), Anderson (2016), Dithmer and Abdulai (2017), Donaldson (2018) and Dithmer and Abdulai (2020)	Decreased food availability because of higher incentives to export	Drèze and Sen (1989), Watts & Bohle (1993), Davis (2000), Devereux (2009) and FAO (2015)
Attenuation of food and non-food price volatility by compensating domestic deficits and shocks	Dorosh et al. (2009), McCorriston et al. (2013) and Rutten et al. (2013)	Higher exposure to economic shocks leading to surge in food and non-food prices	Headey (2011), McCorriston et al. (2013), Rutten et al. (2013), Flachsbarth and Garrido (2014) and Mary (2019)
Lower costs of inputs, higher incentives to invest, and increased agricultural productivity	FAO (2015), Dithmer and Abdulai (2017) and Davalos et al. (2020)	Harms and vulnerability circles for poor rural households	Drèze and Sen (1989), Watts & Bohle (1993), Madeley (2000), Davis (2000), Rosset (2008), Devereux (2009), Díaz-Bonilla (2015)
Economy-wide and sectoral new employment opportunities, higher incomes	Houssa and Verpoorten (2015), Dithmer and Abdulai (2017) and Montalbano et al. (2018)	Increased macroeconomic vulnerability of countries	Davis (2000), Puma et al. (2015), Sartori and Schiavo (2015), Tamea et al. (2016) and Distefano et al. (2018)
Favorable macronutrients’ price differentials on global markets for developing countries	Asche et al. (2015) and Traverso and Schiavo (2020)	Domestic increase of prices for exportable food	FAO (2015)
Stabilization of food and non-food markets; investment attraction	Porteous (2017)	Domestic incomes affected by higher competition	FAO (2015)
Increased diversity of selected micronutrients and macronutrients	Wood et al. (2018) and Traverso and Schiavo (2020)	Lower amount of micronutrients in traded food; diffusion of highly processed, calorie-rich, nutrient-poor food	Blouin et al. (2009), Wood et al. (2018) and An et al. (2019)

Regarding the dimension of *availability*, many authors point out how higher levels of trade openness yield, on average, a better food availability through both increased efficiency in domestic food production and higher quantities of food inflows for importing countries (Burgess & Donaldson, 2010; Dorosh & Rashid, 2013; Baldos & Hertel, 2015; Dithmer & Abdulai, 2017; Donaldson, 2018; Wood et al., 2018; Dithmer & Abdulai, 2020; Traverso & Schiavo, 2020). Trade openness, indeed, appears to favor a more efficient use of resources through specialization, while the higher competition and the availability of cheaper, high-quality inputs can push domestic producers to invest for productivity enhancement (FAO, 2015). Moreover, countries lacking crucial resources, such as land or water, may compensate unmet domestic food demand through food import and may, in turn, specialize in other sectors in which they have a comparative advantage (Anderson, 2016). Food import may also have a positive effect on the stability of domestic food availability in case of negative production shocks, caused for example by extremely disruptive natural events (Dorosh, 2001) or, simply, to compensate for seasonal shortages (FAO, 2015) which often play a main role in undernutrition, particularly among children (Chikhungu & Madise, 2014; Madan et al., 2018). Baldos and Hertel (2015) focus on the role of international food trade in mitigating the food security risks associated to climate change, arguing that integrated markets would contribute to manage short- and long-run effects by increasing food supply in vulnerable areas. On the opposite side, authors that focus on famines with an historical perspective show that, in some circumstances, trade openness led to massive food exports in times when it was needed domestically, therefore negatively affecting food availability (Drèze & Sen, 1989; Watts & Bohle, 1993; Davis, 2000; Devereux, 2009). Indeed, the higher prices in international markets can incentivize domestic producers to divert production from national markets to export (FAO, 2015).

The debate is more controversial for the impact of trade on food *access.* Such impact can emerge not only through direct consequences on the food sector but also through effects on the overall economy, on real incomes and, consequently, on people’s purchasing power (Gries et al., 2009; Kim et al., 2011; Sakyi et al., 2015; Tahir et al., 2014). On the one hand, some scholars observe that trade openness fosters economic wealth and market stability for both food and non-food producers as well as for food consumers, through higher average real incomes resulting from cheaper inputs, greater market access for exports, new employment opportunities, and lower and more stable domestic food and non-food prices (Anderson, 2016; Asche et al., 2015; Davalos et al., 2020; Dithmer & Abdulai, 2017; Dorosh et al., 2009; Houssa & Verpoorten, 2015; McCorriston et al., 2013; Montalbano et al., 2018; Porteous, 2017; Rutten et al., 2013; Traverso & Schiavo, 2020). Dorosh et al. (2009), for example, analyze the production and trade of maize and cassava in Zambia in 2006 and present regional trade as a tool for moderating price volatility domestically. Porteous (2017) studies the impact of maize export bans on prices in five countries in East and Southern Africa, where temporary restrictions have been widely adopted to stabilize domestic prices of staple grains during the food price spike of 2007–2008. Results show that export bans increase prices and price volatility in the implementing countries. Similarly, Bouët and Debucquet (2012) show a worsening of the national welfare for small net food-importing countries in case of a simulation of export taxation and lack of trade policies coordination. Davalos et al. (2020) study the impact of trade openness through the price variation of chemical fertilizers in rural Vietnam, showing that liberalization increased rural household participation into farm employment with direct effects on food availability due to increase productivity and indirect effects on food access through increased farmers’ income. Dorosh and Rashid (2013) deal with the Bangladesh-India rice trade in the 2000s and with the sharp curtail of the exchanges during the 2007 crisis. Through a model simulation, the authors show that openness to trade would have been able to reduce requirements for public stockholding for rice, suggesting therefore a positive effect of trade on both availability and access.

On the other hand many scholars argue that more trade openness increases the exposure of countries to economic shocks, with the consequence of generating both higher and more volatile food prices in food-importing countries and increasing the vulnerability of poor households (Cathie & Herrmann, 1988; Madeley, 2000; Storm, 1999; Rosset, 2008; Béné et al., 2010; Headey, 2011; McCorriston et al., 2013; Rutten et al., 2013; Flachsbarth & Garrido, 2014; Díaz-Bonilla, 2015; Puma et al., 2015; Sartori & Schiavo, 2015; Tamea et al., 2016; Distefano et al., 2018; Mary, 2019). Headey (2011), for example, shows how trade shocks contributed to the surge in food prices in 2008. Analogously, Flachsbarth and Garrido (2014) investigate how different levels of trade openness impacted on international food price transmission to domestic markets in six Latin American countries. They claim that deeper market integration increases global price transmission elasticities, with more agricultural trade openness being associated to higher food Consumer Price Indexes during global price spikes. In addition, trade openness can increase the price of exported products domestically and deteriorate people’s purchasing power, while in import-competing sectors income and employment can be negatively affected by the increased competition (FAO, 2015). Delgado et al. (2005) underline the non-tradability of a high share of staple crops in some African countries, calling therefore for caution in considering the positive income effect derived from trade intensification. Finally, Wobst (2003) identifies that similar trade policy measures may have different impacts on household income and marketing margins depending on the underlying economic structures of the diverse countries.

Regarding food *utilization*, most of the authors find a positive relation between this dimension and trade (Baldos & Hertel, 2015; Traverso & Schiavo, 2020; Wood et al., 2018), highlighting the access to a greater variety of food and a higher compliance with international standards about nutritional guidelines (FAO, 2015). Conversely, Blouin et al. (2009) and An et al. (2019) detect a positive association between trade openness and an increase of obesity and poor diets, with higher consumption of calorie-rich and nutrient-poor aliments after trade liberalization in some developing countries. For this purpose, it is also worth mentioning the results stemming from the recent literature analyzing the effects of trade from a nutritional perspective. Traverso and Schiavo (2020) focus on 71 low-income countries in the period 1996–2014 and analyze the trade evolution of macronutrients (carbohydrates, lipids, and proteins). Their results suggest that the involvement in international food trade has positive effects on low-income countries’ macronutrient availability and access, which can result into improved food utilization. According to these authors, low-income countries, indeed, present a net inflow of all the analyzed macronutrients and benefit from the favorable price differential between exported and imported nutrients. In a similar vein, Wood et al. (2018), by comparing trade versus no-trade scenarios in the period 2007–2011, show that, on average, trade contributes to distribute nutrients among countries and improves countries’ ability to meet their nutritional needs, particularly for macronutrients (i.e. protein and carbohydrates). However, they detect that in several low-income countries trade would actually decrease the availability of some micronutrients.

From the literature review, two lessons can be drawn. First, the overall impact of trade is the outcome of a series of positive and negative effects that can take place at a both economy-wide and sectoral level. On the one hand, indeed, total trade (including food and non-food trade) has an impact on food *access* through both general and sectoral changes in real income; on the other hand, food trade directly affects food *availability* and *utilization* through both food imports and exports. This calls for new research discerning the impacts of total and sectoral trade on the diverse dimensions of food security. Second, such impacts can differ for import and export and, then, they should be studied by differentiating the effects of two sides of trade and by verifying the robustness of general results across different subsamples of countries (net importers vs. net exporters). Our paper contributes to these goals by estimating the relative size of the income-mediated impact of total trade vs. the direct impacts on food security and, then, by focusing on the effects of cereals trade and its two sides (import and export).

## Data and methodology

We measure food security principally focusing on the prevalence of undernourishment, as published by FAO and adopted by both the MDG and SDG initiatives to monitor the global fight against hunger (Goal 1, MDG Indicator 1.9, and Goal 2, SDG Indicator 2.1.1, respectively). In the scientific literature this indicator is often chosen among others in the food security domain (Barrett, 2010; Tiwari & Zaman, 2010; Soriano & Garrido, 2016; Mary, 2019) and is defined as “an estimate of the proportion of the population whose habitual food consumption is insufficient to provide the dietary energy levels that are required to maintain a normal active and healthy life” (UN, 2019). Probably due to its prominence in the development agendas, the time series for this key indicator were reconsidered and updated over time for many countries; as a consequence, the most recently published data only cover the period from 2001 (average 2000–2002) and are not reconciled with previous time series. This makes our panel smaller and not exactly the same as that used by other authors previously. We also perform robustness tests by alternatively measuring food security through the daily average energy intake (dietary energy consumption). This is the main variable used by Dithmer and Abdulai (2017) and nonetheless it is a less preferred option for us, as it does not account for distributional issues that are hidden beyond average values (Mary, 2019) and, then, for the actual access to food by the poor.

**Fig. 2:**
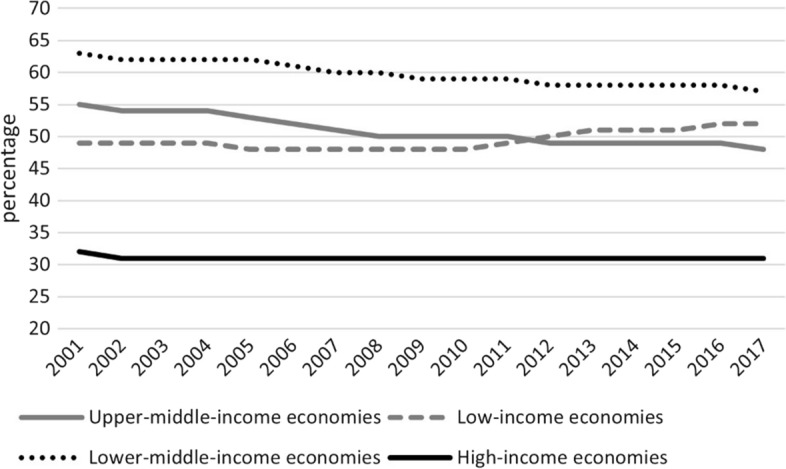
Share of dietary energy supply derived from cereals, roots and tubers (kcal/cap/day) (3-year average). *Source*: FAO (2022)

Trade openness is measured by the sum of a country's exports and imports as a percentage of GDP. For total trade, we take data from the World Development Indicators (WDI) published by the World Bank. In line with this definition of trade openness and following the extant literature that proposes a sector-level notion of trade openness (Di Giovanni & Levchenko, 2009; Flachsbarth & Garrido, 2014; Mary, 2019), we also construct a measure of trade openness for the cereals subsector, based on cereals’ import, export and production values published by FAO; analogously, we calculate complementary measures of trade openness for sectors other than cereals (i.e. non-cereals trade as a percentage of non-cereals GDP). The choice of focusing on cereals trade is based on three considerations: the importance of staple food for protein and energy undernutrition, which is still a main challenge for developing countries (Traverso & Schiavo, 2020) notwithstanding recent emphasis on micronutrients and hidden hunger (Wood et al., 2018); the role of trade in linking food-surplus areas with food-deficit areas, which is particularly relevant for staples, also thanks to cereals’ shelf-life (Brooks & Mattews, 2015; Wright, 2012); the centrality of cereals in recent export bans (Porteous, 2017).^4^ Figure 2 shows that the contribution of cereals to people’s energy intake is much higher in low and middle countries than in high income countries and that it is also rising in low-income ones, where a balanced, diversified diet remains beyond the reach of many, particularly among the poor.

We further decompose this measure into import openness and export openness since the two sides of trade can have diverging effects on food security, as discussed in Sect. 2. All the trade openness variables enter our models as natural logarithms (linear-log model) reflecting diminishing marginal returns of trade openness on food security; as already pointed out by Mary (2019), it is indeed reasonable to expect that, as trade openness increases, its effect on hunger becomes smaller.

Following the available literature (Dithmer & Abdulai, 2017; Mary, 2019), we recognize potential endogeneity of trade openness which might result from reverse causality and from the possibility that governments and market actors change their choices about international trade according to the levels of food insecurity in their countries. This problem was addressed by Dithmer and Abdulai (2017) by adopting a GMM estimator. This estimator however requires large samples in the cross-section dimension (Arellano & Bond, 1991) and then may not be appropriate for cross-country datasets that often have a small number of units (Bond, 2002; Panizza & Presbitero, 2014), like in our case. Moreover, the use of lagged levels of the explanatory variables as internal instruments has been highly questioned, especially when the underlying process is persistent over time and the lag length is short (Bellemare et al., 2017; Moral-Benito, 2013; Moral-Benito, 2013; Panizza & Presbitero, 2014). Conversely, Mary (2019) adopts an empirical strategy based on the instrumental variable approach but, failing to find a proper instrument for trade openness, ends up modelling the effect of food security (instrumented by rainfall anomalies) on trade openness and, then, using the residual trade openness that is not explained by food security as an instrument in his main model.

Taking inspirations from the extant literature, we instead orient our efforts toward the identification of an instrumental variable for trade openness that complies with the exogeneity and relevance requirements. In their seminal work on the impact of trade openness on economic growth, Frankel and Romer (1999) suggest that countries’ geographic characteristics have important effects on trade and employ them as valid instruments. However, in our case, geographic factors may also be correlated with food security, as they can affect agricultural production and yields. Moreover, obtaining time-variant geographic variables, which is necessary for panel data analysis, and adapting such instruments to be sector specific is challenging. Romalis (2007) then proposes an alternative instrument for trade openness by taking the US Most Favored Nation tariff rates and recognizing that how internationally integrated a country is depends both on its own policies and on the policies of its trading partners. In other words, countries would become more exposed to trade when also the trading partners liberalize their trade regimes. Based on this intuition, we assume that the openness of trade partners and, more generally, the average trade openness of the world affect the exposure to trade of each country, as also shown by the recent trends during and after the Great Recession (UNCTAD, 2016, 2017; WTO, 2017). However, an instrument based on the average trade openness of the world would be affected by two problems: first, it would be also influenced by the level of trade openness of each country in the sample, thus violating the exogeneity requirements; second, it would be country-invariant and thus not suitable for panel data models. For these reasons, for each country (and year), we define and construct our instrument for trade openness as the trade openness of the rest of the world. In line with the definition of trade openness provided above, this is done by taking the aggregate world value of imports, exports and production, as provided by the respective databases (World Bank WDI for the total economy and FAO for cereals), and subtracting from each the corresponding value for the country. The proposed instrument has the advantage of being both country- and time-variant and sector-specific where necessary. We verify the endogeneity of trade openness by performing both Hausman’s (1978) and Davidson–MacKinnon’s (1981) tests, while the relevance of the instrument is assessed by means of the Anderson-Rubin Wald test (Anderson & Rubin, 1949). Following Biener et al. (2020), we also try to detect possible correlation between the instrument and unmeasured variables potentially affecting undernutrition, thus violating the exclusion restriction. Potentially, indeed, trade openness of foreign countries could be correlated to other dimensions of their participation in international flows, such as foreign direct investment (FDI) and official development assistance (ODA), which in turn may partially affect food security. In our sample, both FDI and ODA show a negligible correlation with trade openness of the rest of the world (ρ close to zero for all instruments). To further mitigate remaining concerns that the instrument is correlated with unobservables in the error term, and based on Biener et al. (2020)’s intuition that controlling for the observed covariates in the model can mitigate any potential correlation with unobservable factors, we perform a robustness test by adding the two covariates, FDI and ODA, to the regressors of our main model and obtain full confirmation of the results (Appendix 3, Table 10, Column 12).

Our model specification, in the general form, is a linear-log with fixed effects and cluster robust standard errors, as presented in Eq. (1):

1 $$U_{{it}} = \alpha + \mathop \sum \limits_{h}^{H} \beta _{h} \ln TO_{{it}} + \mathop \sum \limits_{k}^{K} \gamma _{k} X_{{k,it}} + \eta _{i} + u_{{it}}$$

where U_it_ is the percentage of undernourished in country *i*’s population and TO_it_ is the (instrumented) trade openness. Trade openness measures are based on data on trade and production from FAOSTAT as detailed in Appendix 2.^5^ The resulting variables are: total trade openness (TTO_it_); cereals trade openness (CTO_it_) and non-cereals trade openness (NCTO_it_); cereals import openness (CIO_it_) and cereals export openness (CEO_it_), as written in Eqs. (2), (3), and (4):

2 $$U_{{it}} = \alpha + \beta _{1} \ln (TTO)_{{it}} + \mathop \sum \limits_{k}^{K} \gamma _{k} X_{{k,it}} + \eta _{i} + u_{{it}}$$

3 $$U_{{it}} = \alpha + \beta _{1} ln\left( {CTO} \right)_{{it}} + \beta _{2} {\text{ln}}(NCTO)_{{it}} + \mathop \sum \limits_{k}^{K} \gamma _{k} X_{{k,it}} + \eta _{i} + u_{{it}}$$

4 $$U_{{it}} = \alpha + \beta _{1} \ln \left( {CIO} \right)_{{it}} + \beta _{2} \ln \left( {CEO} \right)_{{it}} + \beta _{3} \ln (NCTO)_{{it}} + \mathop \sum \limits_{k}^{K} \gamma _{k} X_{{k,it}} + \eta _{i} + u_{{it}}$$

In all specifications, X_it_ is the same set of control variables, which is omitted in the baseline models (models a), or limited to one variable (GDP per capita) in intermediate models (models b). Finally, *u*_*it*_ is the cluster robust error term. Our selection of control variables largely relies on Dithmer and Abdulai (2017) and Mary (2019) and their expected effect on undernourishment are briefly described below, while data sources and description are provided in Appendix 2.

*Real GDP per capita* is taken to represent the average income and how changes in the economic environment can affect people’s purchasing power and, consequently, food security through economic access to food. *Population growth* is expected to create demographic pressure and affect per capita food availability, thus increasing the prevalence of undernourishment. The percentage of *rural population* reflects the structure of the economy and also represents a proxy for living standards, which are often lower in rural areas. For this reason, we expect rural population to be positively related to undernourishment. *Average cereal yields* are a measure of physical productivity of agriculture, reflecting intensive growth of the sector, which is expected to positively affect food security. *Arable land*, as a percentage of total land area, reflects the agricultural potential of a country and the extensive growth of the agricultural sector, both of which, ceteris paribus, should improve food security. *Inflation* based on Food Consumer Price Index is expected to be detrimental to consumers’ food access. Unfortunately, this index is not available specifically for cereals. On the contrary, inflation based on Cereals Production Price Index could contribute to increase income and to reduce food insecurity of cereals producers, i.e. a large part of rural population in developing countries, but it is also collinear with inflation based on consumer prices, making the direction of the prevailing effect uncertain. The share of population affected by *natural disasters*, calculated as a percentage of total population, is of course expected to negatively impact on food security. A variable for the *occurrence of wars* (major episodes of political violence) is also expected to have negative impacts on food security.

The coefficient for total trade openness that is estimated in Eq. 2 represents the *direct* impact that the variable has on food security. However, besides this effect, total trade openness can also affect income in all sectors of economic activity, which in turn is expected to affect food security in terms of economic access to food. This *indirect* effect of total trade openness and its relative importance are estimated by applying Smith and Haddad’s multi-step methodology (Smith & Haddad, 2002). The first step consists in regressing the prevalence of undernourishment on trade openness excluding the GDP per capita from the set of control variables. This allows the identification of the *total* effect of trade openness on food security, also including the effect that passes through income. In order to improve the explanatory power of the model and better isolate the effect of (instrumented) trade openness, we include the full set of control variables.

5 $$U_{it} = \alpha + \beta_{1} lnTTO_{it} + \mathop \sum \limits_{k}^{K - 1} \gamma_{k} X_{k,it} + \eta _{i} + u_{it}$$

The second step aims at assessing the existence of a significant association between food security and the intermediate variable by estimating the following equation:

6 $$U_{it} = \alpha + \mathop \sum \limits_{k}^{K} \gamma_{k} X_{k,it} + \eta _{i} + u_{it}$$

The third step estimates the effect of (instrumented) trade openness on per capita income:

7 $$GDP_{it} = \alpha + \beta_{1} lnTTO_{it} + \mathop \sum \limits_{k}^{K - 1} \gamma_{k} X_{k,it} + \eta _{i} + u_{it}$$

Like Eq. 2, all the above equations are estimated using fixed-effects regressions. By combining the estimates of Eq. 6 with those obtained from Eq. 7, we are then able to calculate the *indirect* effect of trade openness that passes through income as follows:

8 $${\text{Indirect}}\,{\text{effect}}\,{\text{of}}\,{\text{TTO}}\,{\text{through}}\,{\text{GDP}} = \left( {\frac{\partial U}{{\partial GDP}}} \right) \times \left( {\frac{\partial GDP}{{\partial TTO}}} \right)$$

Our sample includes all the observations corresponding to countries classified as low-income economies or middle-income economies by the World Bank for the reference year.^6^ The dimension of the sample also depends on the availability of the explanatory and control variables. In order to keep the sample comparable across the different models we use the sample corresponding to the model with the highest number of independent variables, i.e. the smallest sample. This sample includes 81 countries and 1021 observations (the list of countries in the sample is provided in Appendix 1).

## Results

### Total trade openness and undernourishment

Table 2 presents the results for the baseline (2a), intermediate (2b) and full (2c) models corresponding to Eq. (2), where trade openness is measured by total trade. Trade openness is confirmed to be endogenous by the Davidson–MacKinnon’s test and by the Hausman test, which compares model (2c) with the corresponding model estimated without the use of instrumental variables (2d). The adopted instrument, which is the total trade openness of the rest of the world, is found to be significant in the first stage regressions, and the Anderson–Rubin Wald test confirms its relevance.^7^

**Table 2:** Total trade openness and undernourishment

	2SLS-IV FE			OLS FE
	(2a)	(2b)	(2c)	(2d)
Total trade openness (log)	− 33.4645 (− 3.09)***	− 16.0321 (− 2.99)***	− 11.9001 (− 2.52)**	1.3516 (2.16)**
GDP per capita (constant USD 2010)		− 0.0012 (− 3.40)***	− 0.0008 (− 2.11)**	− 0.0007 (− 6.67)***
Population growth			0.1696 (0.68)	0.2259 (1.46)
Rural population (%)			0.2256 (1.69)*	0.2775 (6.49)***
Cereal yield (kg per ha)			− 0.0002 (− 0.54)	− 0.0006 (− 3.04)***
Arable land (% of land area)			− 0.4643 (− 3.06)***	− 0.3381 (− 5.35)***
Inflation based on Food Consumer Price Index			2.7608 (1.19)	− 1.9309 (− 1.46)
Inflation based on Cereals Production Price Index			0.7515 (1.40)	− 0.1899 (− 0.52)
Natural disasters			− 1.7916 (− 0.52)	1.0757 (0.54)
Conflicts			0.4741 (1.13)	0.1870 (1.32)
Constant				3.5392 (0.90)
N of countries	81	81	81	81
N of observations	1021	1021	1021	1021
F	9.42***	9.41***	4.07***	29.30***
Hausman test (*p* value)			0.000	
Anderson− Rubin Wald test (Chi squared p value)	0.0000	0.0000	0.0016	
Davidson− MacKinnon test F (*p* value)	0.0000	0.0000	0.0000	

Robust z statistic in parentheses

***p < 0.01; **p < 0.05; *p < 0.1

The coefficient for trade openness is negative and significant but becomes smaller as the other relevant regressors are added. From the full model, it emerges that a 1% increase (decrease) in trade openness decreases (increases) the prevalence of undernourishment by around 0.12 points. If applied to the average prevalence of undernourishment in the sample, this corresponds to a 1.07% decrease (increase).^8^ In line with our expectations, per capita income and the share of arable land are significantly and negatively correlated with undernourishment, while the share of rural population significantly and positively. Conversely, the coefficients of the remaining variables are not significant. In the case of natural disasters and conflicts, statistical insignificance can be due to the local impact of some of these events, while our dependent variable is at the national level.

### Income-mediated and direct effects of total trade openness on undernourishment

When calculating the direct and income-mediated effects of trade openness on undernourishment, as explained in Sect. 3 and detailed in Tables 3 and 4, we find that the effect of trade openness which passes through income (*indirect* effect) accounts for around 37% of total effect, while 63% of the effect is *direct*. In other words, most of the estimated effects of trade are not mediated by generalized changes in real income, which influence economic *access* to food, but would directly impact the other dimensions of food security (*availability*, *utilization* and *stability*). This seems to suggest that trade would mainly have effects through its food component, while the economy-wide impacts on undernourishment would be smaller. In front of a sizable association between trade and GDP per capita (step 3 in Table 3), this result is mainly due to the very small effect that per capita income seems to have on the prevalence of undernourishment (coefficient close to 0). While the low relevance of income in determining undernourishment may seem counterintuitive, it should be noticed that this variable does not account for distributional issues, which conversely have been considered as determinant factors by the extant literature on food security (Behrman & Deolalikar, 1987; Brown et al., 2019; Haddad et al., 1996; Smith & Haddad, 2002; Sorriano & Garrido, 2016).^9^

**Table 3:** Estimates of direct and indirect effects of total trade openness on undernourishment (2SLS- IV FE)

	Step 1	Step 2	Step 3
	Dep var: undernourishment	Dep var: undernourishment	Dep var: GDP pc
GDP pc		− 0.0007**	
Total trade openness (log)	− 19.7693***		9421.844***
Countries	81	81	81
Obs	1021	1021	1021
Anderson- Rubin wald test (*p* value)	0.0000	No IV	0.0000

Robust z statistic in parentheses

***p < 0.01; **p < 0.05; *p < 0.1. Full results are available upon request

**Table 4:** Decomposing the direct and indirect effects of total trade openness on undernourishment

	Total effect	Direct effect	Indirect effect through per capita GDP
Value	− 18.9703	− 11.9001	− 7.07015
%	100%	63%	37%

Own elaborations on coefficients reported in Tables 2 (Column 2c) and 3 (Columns 1, 2, 3)

### Cereals trade openness and undernourishment

In line with the hypothesis that economy-wide impacts do not play a main role in the relationship between trade and undernourishment, we focus on the component of trade that is more likely to have direct effects on food security, that is, as discussed before, cereals trade. For this purpose, Table 5 reports the estimated coefficients for Eq. (3), where cereals are taken out from total trade openness and trade openness for all products other than cereals is also included. Cereals and non-cereals trade openness are confirmed to be endogenous by the Davidson–MacKinnon’s test and by the Hausman test. The instruments, which are respectively cereals and non-cereals trade openness of the rest of the world, are found to be significant in the first stage regressions, with the Anderson–Rubin Wald test confirming their joint significance.

**Table 5:** Cereals trade openness and undernourishment

	2SLS-IV FE			OLS FE
	(5a)	(5b)	(5c)	(5d)
Cereals trade openness (log)	− 11.8712 (− 3.08)***	− 11.9341 (− 2.23)**	− 6.8324 (− 2.55)**	− 0.0869 (− 0.43)
Non-cereals trade openness (log)	− 2.2254 (− 0.36)	− 2.2485 (− 0.40)	− 2.8927 (− 0.72)	1.2950 (2.07)**
GDP per capita (constant USD 2010)		0.0000 (0.02)	− 0.0001 (− 0.15)	0.0007 (− 6.44)***
Population growth			− 0.1627 (− 0.45)	0.2196 (1.41)
Rural population (%)			0.1548 (0.84)	0.2753 (6.42)***
Cereal yield (kg per ha)			− 0.0010 (− 1.56)	− 0.0006 (− 3.07)***
Arable land (% of land area)			− 0.5336 (− 2.98)***	− 0.3416 (− 5.40)***
Inflation based on Food Consumer Price Index			3.8111 (1.79)*	− 1.8707 (− 1.41)
Inflation based on Cereals Production Price Index			− 1.2649 (− 1.43)	0.2203 (− 0.59)
Natural disasters			3.6903 (0.92)	1.1484 (0.57)
Conflicts			0.3879 (1.25)	0.1900 (1.34)
Constant				4.2381 (1.08)
N of countries	81	81	81	81
N of observations	1021	1021	1021	1021
F	5.62***	3.92**	3.25***	26.56***
Hausman test (*p* value)			0.0037	
Anderson–Rubin Wald test (Chi squared *p* value)	0.0000	0.0000	0.0001	
Davidson–MacKinnon test F (*p* value)	0.0000	0.0000	0.0000	

Robust z statistic in parentheses

***p < 0.01; **p < 0.05; *p < 0.1

Interestingly enough, the coefficient for non-cereals trade openness is not significant, while the coefficient for cereals trade openness is negative and significant. Cereals trade openness, then, seems to drive the positive effects of total trade openness on undernourishment and this is coherent with the predominance of the effects of trade which do not pass through economy-wide changes in income, as found in Table 4. The size of the coefficient suggests that, when cereals trade openness increases (decreases) by 1%, the prevalence of undernourishment decreases (increases) by about 0.07 points, corresponding to a 0.6% change if calculated on the sample average value.^10^ Most of the other coefficients maintain the expected sign, although only the share of arable land and the inflation based on Food Consumer Price Index turn out to be significantly associated to the dependent variable. In particular, the coefficient of GDP per capita remains very small and loses its statistical significance, confirming that the changes in the average income, which does not account for distributional issues, do not have much impact on the prevalence of undernourishment.

### Cereals import, cereals export and undernourishment

As cereals trade turns out to be a dominant factor in leading the impacts of trade openness on undernourishment, we further investigate the channels through which it occurs by decomposing cereals trade into exports and imports, instrumented respectively by cereals imports and exports of the rest of the world (Table 6). Also in this case, the instrumented variables are confirmed to be endogenous by the Davidson–MacKinnon’s test, while the Hausman test, performed on the coefficients of model 6c and of the corresponding OLS model (6d), does not reject the non-instrumented version, which, in any case, yields similar sign and significance of the coefficients under analysis. The adopted instruments are found to be statistically significant in the first stage regressions (only in model 6a the instrument for cereals exports is not significant) and the Anderson-Rubin Wald test validates their joint significance in all models.

**Table 6:** Cereals import, cereals export and undernourishment

	2SLS-IV FE			OLS FE
	(6a)	(6b)	(6c)	(6d)
Cereals import openness (log)	− 17.9606 (− 2.46)**	− 12.3234 (− 1.94)*	− 9.4898 (− 2.06)**	− 0.3707 (− 1.98)**
Cereals export openness (log)	2.1900 (0.36)	2.2535 (0.50)	1.6067 (0.64)	0.0342 (0.49)
Non-cereals trade openness (log)	− 2.2416 (− 0.17)	− 1.2631 (− 0.14)	− 1.8311 (− 0.30)	1.4046 (2.25)**
GDP per capita (constant USD 2010)		− 0.0009 (− 0.97)	− 0.0006 (− 0.94)	− 0.0007 (− 6.53)***
Population growth			− 0.2979 (− 0.63)	0.2055 (1.32)
Rural population (%)			− 0.0977 (− 0.37)	0.2628 (6.07)***
Cereal yield (kg per ha)			− 0.0023 (− 1.96)*	− 0.0007 (− 3.36)***
Arable land (% of land area)			− 0.5735 (− 2.70)***	− 0.3472 (− 5.50)***
Inflation based on Food Consumer Price Index			4.2308 (1.13)	− 1.7411 (− 1.31)
Inflation based on Cereals Production Price Index			− 0.3494 (− 0.19)	− 0.2404 (− 0.65)
Natural disasters			5.0683 (0.88)	1.2977 (0.65)
Conflicts			0.8918 (1.70)*	0.2066 (1.45)
Constant				5.4865 (1.39)
N of countries	81	81	81	81
N of observations	2021	2021	1021	1021
F	2.17*	2.70**	2.44***	24.74***
Hausman test (*p* value)			0.417	
Anderson–Rubin Wald test (Chi squared *p* value)	0.0000	0.0000	0.0000	
Davidson–MacKinnon test F (*p* value)	0.0000	0.0000	0.0000	

Robust z statistic in parentheses

***p < 0.01; **p < 0.05; *p < 0.1

The coefficient for cereals imports is negative and statistically significant, reflecting the positive effect that this side of cereals trade has on undernourishment; conversely, the coefficient for cereals exports is not significant. Such results seem coherent with what emerged in previous regressions. Since trade was found to have a less relevant impact on the economic *access* to food, i.e. through income, and to mainly affect food security through direct effects on the other dimensions, it is reasonable to expect these effects being principally led by imports. In fact, while exports would mainly benefit cereals exporters through improvements in their income (thus affecting *access* to food), a greater openness to cereals imports is supposed to especially bring advantages to consumers by improving food availability and stability, as also discussed in Sect. 2. In other words, the positive direct effects of trade openness on undernourishment that we detect would be largely due to the improved availability, stability and utilization of food which are ensured by international trade and, particularly, by the import of cereals, which would contribute to compensate both structural and temporary domestic food deficits, to smooth seasonality and fluctuations in staple food availability, to reduce countries’ vulnerability to supply-side shocks and to strengthen the resilience of food systems.

### Robustness tests

In order to make sure that our findings are not driven by particular groups of countries or observations, we finally perform a series of robustness tests on selected subsamples. Results for the coefficient of cereals trade, based on the full model (Eq. 3), are summarized in Table 10 of Appendix 3. First, in line with previous considerations, we divide the sample between net importers and exporters of cereals. While the statistical significance and the negative sign of the coefficient of cereals trade are confirmed in the subsample of net cereals-importing countries, no significant results emerge from the subsample of net exporters which, however, is much smaller,^11^ in line with the actual distribution of countries (Ng & Aksoy, 2008). This would also confirm that the main effects of cereals trade on undernourishment principally occur through imports.

Second, we alternatively exclude from the sample: the outliers, detected through the Hadi procedure (Hadi, 1992); the ten countries with the most extreme values of cereals trade; the sixteen countries with the most extreme values of undernourishment prevalence^12^; countries from specific regions (South Asia, East Asia and Pacific, Sub-Saharan Africa, Latin America, Middle East and North Africa, Europe and Central Asia). In all these cases, our results appear robust.

Finally, we estimate Eqs. 2 and 3 by employing the average energy intake per capita as alternative dependent variable (Table 11, Appendix 3). Also in this case, our main results remain robust. It should be noticed that also the coefficients for non-cereals trade openness and per capita income turn out to be positive and significant, while the same were not significant when food security was measured by the prevalence of undernourishment. As already underlined, the average energy intake per capita does not capture how food is distributed, as it is instead reflected in the prevalence of undernourishment; analogously, GDP per capita does not tell anything about how income is distributed. This suggests that non-cereals trade has an effect on the average level of food security, which may also pass through increases in per capita income. However, this seems limited to an increase in the average food consumption which does not significantly impact on the poorest, who are the undernourished.

## Conclusions and policy implications

This study provides new evidence about the effects of international trade on undernourishment for the period 2001–2016 and points out three main conclusions. *First*, trade openness contributes to lower the prevalence of undernourishment in developing countries and most of this effect does not pass through the increases that it produces in average income at an economy-wide level (i.e. affecting the economic access to food) but, rather, through the impacts that it has on the other dimensions of food security. *Second*, such impacts are mostly driven by the trade openness of the cereals sector where, *third*, the import component turns out to play the main role.

These results seem to support Dithmer and Abdulai (2017), who find that total trade openness is positively related to average dietary energy consumption, rather than Mary (2019). However, the positive relationship between trade openness and food security that emerges here is narrower, being mainly based on the role of cereals import, and does not allow to extend the narrative about the success of free trade in the fight against hunger to the other sectors. Our findings indeed suggest that import openness in the cereals sector improves the nutritional status of the poorest and the resilience of countries’ food systems to internal shocks in food production, hence supporting the nexus between trade openness and food availability (and its stability) described in the literature (Baldos & Hertel, 2015; Burgess & Donaldson, 2010; Donaldson, 2018; Dorosh & Rashid, 2013; FAO, 2015; Traverso & Schiavo, 2020; Wood et al., 2018), while trade in sectors other than cereals does not appear to have any significant effect.

It is worth underlining that the beneficial effect of cereals import holds even after controlling for the main determinants of both domestic food production (like extensive and intensive growth of the agricultural sector) and economic welfare in the country (per capita income and food prices). The results are confirmed also in the subsample of net cereals-importing countries, i.e. countries where the production capacity is below actual consumption, which are the majority of low and middle countries countries. This is a key issue when interpreting our results. Increasing import openness as it is measured here, basically, means that import has to grow faster than production, but is does not mean that production should not grow as well. This would be non-sense and the sign and significance of coefficients of variables that measure agricultural extension and productivity are clear about this. The point is probably that countries that are successful in their fight against undernutrition do not only increase their domestic production, but they also allow import to grow even faster to improve domestic food availability and its stability.

In terms of policy implications, this confirms that the polarized debate about food self-sufficiency as the opposite of international trade is not appropriate, as suggested by Clapp (2017), who calls for a more nuanced approach. If self-sufficiency is defined as the ratio of production to consumption, where consumption is production plus net trade, policy makers would better focus on the numerator, pursuing the increase of domestic production, and allow trade (the denominator) to compensate for remaining gaps from actual needs.

These conclusions support the evidence that trade restrictions in the cereals sector may seriously hurt developing countries (Porteous, 2017) and this is particularly meaningful today, as the resurgence of protectionism (Fajgelbaum et al., 2020) has been further exacerbated by the export restrictions that many countries have implemented as a reaction to the shocks caused by the Covid-19 pandemic (FAO, 2020; Kerr, 2020; WTO, 2020). Using the estimated coefficient for total trade openness (Table 2, column 2c) and employing the pre- and post-Covid19 IMF trade projections for the group of developing countries (October 2019 and October 2020, respectively), it is possible to calculate, *ceteris paribus*, the change in the prevalence of undernourishment caused by the pandemic.^13^ This corresponds to an increase of 0.73 points, i.e. to almost 47 million of new undernourished in 2020 as a consequence of the global outbreak. While these are only preliminary and out-of-sample estimates, they clearly call for new research studying the actual short- and long-term impact of the recent drop in international trade and the food policies that should be implemented to contain the consequences on food security which could further undermine the achievement of the ‘*Zero Hunger*’ Goal.

## Data Availability

All datasets utilized in this study are publicly available at the sources listed in Table 8 in Appendix 2.

## Appendix

### Appendix 1: List of countries in the sample

See Table 7.

**Table 7:** List of countries in the sample

Albania	Colombia	Iran, Islamic Rep	Mozambique	Slovak Republic
Algeria	Congo, Rep	Iraq	Namibia	South Africa
Argentina	Costa Rica	Jordan	Nepal	Sri Lanka
Armenia	Croatia	Kazakhstan	Nicaragua	Suriname
Azerbaijan	Czech Republic	Kenya	Nigeria	Tanzania
Bangladesh	Ecuador	Kyrgyz Republic	Pakistan	Thailand
Belarus	Egypt, Arab Rep	Latvia	Panama	Togo
Benin	El Salvador	Lebanon	Paraguay	Tunisia
Botswana	Estonia	Lithuania	Peru	Turkey
Brazil	Ethiopia	Madagascar	Philippines	Ukraine
Bulgaria	Gambia, The	Malawi	Poland	Uruguay
Burkina Faso	Georgia	Malaysia	Romania	Venezuela, RB
Cambodia	Ghana	Mali	Russian Federation	Vietnam
Cameroon	Honduras	Mauritius	Saudi Arabia	
Chad	Hungary	Mexico	Senegal	
Chile	India	Mongolia	Serbia	
China	Indonesia	Morocco	Sierra Leone	

### Appendix 2: Variables and descriptive statistics

See Tables 8 and 9.

**Table 8:** List of variables and sources

Variable	Description	Source
Prevalence of undernourishment	Prevalence of undernourishment (percent) (3-year average)	Code: 210041 http://www.fao.org/faostat/en/#data/FS
Dietary energy consumption	Dietary energy supply used in the estimation of the prevalence of undernourishment (kcal/cap/day) (3-year average)	Code: 22000 www.fao.org/faostat/en/#data/FS
Total trade openness	Calculated from exports and imports of goods and services and GDP in current USD from WDI	databank.worldbank.org/source/world-development-indicators Exports of goods and services (current US$) [NE.EXP.GNFS.CD] Imports of goods and services (current US$) [NE.IMP.GNFS.CD] GDP (current US$) [NY.GDP.MKTP.CD]
Rest of the world trade openness	Calculated by subtracting individual countries imports and exports from the world’s imports and exports (numerator) and individual countries’ GDP from world GDP (denominator)	
Cereals trade openness	Import and export values and value of production are both from FAO	www.fao.org/faostat/en/#data/TP now TCL (Trade, crops and livestock products) www.fao.org/faostat/en/#data/QV (1000 United States dollars) (Production, Value of Agricultural Production) Gross Production Value (current million US$)
Non-cereals trade openness	Calculated by subtracting cereals’ import and export values (FAO) from total trade (WDI) at numerator and by subtracting cereals production value (FAO) from GDP (WDI) at denominator	www.fao.org/faostat/en/#data/TP now TCL www.fao.org/faostat/en/#data/QV databank.worldbank.org/source/world-development-indicators
Rest of the world non-cereals trade openness	Calculated from the same FAO and WB data introduced above	www.fao.org/faostat/en/#data/TP now TCL www.fao.org/faostat/en/#data/QV databank.worldbank.org/source/world-development-indicators
Cereals’import openness	Import values and value of production are both from FAO	www.fao.org/faostat/en/#data/TP now TCL www.fao.org/faostat/en/#data/QV
Cereals’export openness	Export values and value of production are both from FAO	www.fao.org/faostat/en/#data/TP now TCL www.fao.org/faostat/en/#data/QV
Rest world cereals trade openness	Import and export values and value of production are both from FAO	www.fao.org/faostat/en/#data/TP now TCL www.fao.org/faostat/en/#data/QV
Rest of the world cereals’s import openness	Import values and value of production are both from FAO	www.fao.org/faostat/en/#data/TP now TCL www.fao.org/faostat/en/#data/QV
Rest of the world cereals’ export openness	Export values and value of production are both from FAO	www.fao.org/faostat/en/#data/TP now TCL www.fao.org/faostat/en/#data/QV
GDP per capita (constant USD 2010)	The World Bank World Development Indicators	databank.worldbank.org/source/world-development-indicators
Population growth	The World Bank World Development Indicators	databank.worldbank.org/source/world-development-indicators
Rural population (%)	The World Bank World Development Indicators	databank.worldbank.org/source/world-development-indicators
Cereal yield (kg per ha)	The World Bank World Development Indicators	databank.worldbank.org/source/world-development-indicators
Arable land (% of land area)	The World Bank World Development Indicators	databank.worldbank.org/source/world-development-indicators
Inflation based on Food Consumer Price Index	We calculate the annual rate of change of the price index by averaging FAO monthly food consumer price index	www.fao.org/faostat/en/#data/CP
Inflation based on Cereals Production Price Index	We calculate the annual rate of change of FAO annual cereals production prices index	www.fao.org/faostat/en/#data/PP
Natural disasters	Share of population affected by natural disasters calculated by dividing the number of people affected from EM-DAT by the population (World Bank World Development Indicators)	EM-DAT, The international Disaster Database is an initiative of the Centre for Research on the Epidemiology of Disasters—CRED of the Université Catholique de Louvain. www.emdat.be/about
Conflicts	Magnitude score of major episodes of political violence	MAJOR EPISODES OF POLITICAL VIOLENCE (MEPV) AND CONFLICT REGIONS, 1946–2018 Monty G. Marshall, Center for Systemic Peace July 25, 2019. www.systemicpeace.org/inscrdata.html
Foreign direct investments	FDI net inflows (% of GDP)	databank.worldbank.org/source/world-development-indicators [BX.KLT.DINV.WD.GD.ZS]
Net official development assistance	Net ODA (% of GNI)	databank.worldbank.org/source/world-development-indicators [DT.ODA.ODAT.GN.ZS]

**Table 9:** Descriptive statistics

Variable	Obs	Mean	Std. Dev	Min	Max
Prevalence of undernourishment	1021	11.08	8.85	2.50	40.20
Dietary energy consumption	901	2756.74	382.14	1933.00	3657.00
(Log) Trade openness	1021	4.26	0.44	3.08	5.35
(Log) Rest of the world trade openness	1021	4.00	0.10	3.82	4.13
(Log) Cereals trade openness	1021	3.95	1.63	− 0.28	13.28
(Log) Non-cereals trade openness	1021	4.28	0.44	3.05	5.35
(Log) Rest of the world cereals trade openness	1021	3.38	0.13	3.19	3.78
(Log) Rest of the world non-cereals trade openness	1021	4.01	0.10	3.83	4.14
(Log) Cereals’ import openness	1021	3.34	2.08	− 5.46	13.20
(Log) Cereals’ export openness	1021	0.66	3.02	− 9.73	10.69
(Log) Rest of the world cereals’ import openness	1021	2.74	0.12	2.56	3.11
(Log) Rest of world cereals’ export openness	1021	2.64	0.13	2.40	3.06
GDP per capita (constant USD 2010)	1021	4720.17	3822.68	334.48	18,034.72
Population growth	1021	1.34	1.30	− 9.08	7.79
Rural population (%)	1021	45.48	19.56	5.39	86.05
Cereal yield (kg per ha)	1021	2761.00	1467.84	217.60	9453.70
Arable land (% of land area)	1021	17.49	14.40	0.29	63.79
Inflation based on Food Consumer Price Index	1021	0.09	0.18	− 0.38	1.69
Inflation based on Cereals Production Price Index	1021	0.10	0.26	− 0.67	2.36
Natural disasters	1021	0.02	0.05	0.00	0.44
Conflicts	1021	0.54	1.41	0.00	9.00

### Appendix 3. Robustness tests

See Tables 10 and 11.

**Table 10:** Coefficient of (log) cereals trade openness (model 5c, Table 5)

	Net exporters only	Net importers only	Without under- nourishment extremes	Without cereals trade openness extremes	Without outliers	Without South Asia	Without East Asia and Pacific	Without SSA	Without LAC	Without MENA	Without Europe and Central Asia	Controlling for FDI and ODA
	1	2	3	4	5	6	7	8	9	10	11	12
Cereals trade openness (log)	− 8.4560 (− 0.43)	− 7.3367 (− 2.48)**	− 6.3912 (− 2.36)**	− 8.6082 (− 2.11)**	− 6.1808 (− 2.76)***	− 6.1569 (− 2.19)**	− 7.0021 (− 2.42)**	− 3.7851 (− 2.10)**	− 8.9225 (− 2.11)**	− 8.5717 (− 2.30)**	− 7.3775 (− 2.51)**	− 6.8593 (− 2.52)**
N of countries	24	66	57	71	80	76	73	59	65	72	60	69
N of observations	255	788	737	881	1005	953	910	751	821	899	771	791
Anderson–Rubin Wald test (Chi squared *p* value)	0.2366	0.0001	0.0015	0.0010	0.0001	0.0014	0.0002	0.0216	0.0000	0.0000	0.0001	0.0004

Robust z statistic in parentheses

****p* < 0.01; ***p* < 0.05; **p* < 0.1. Full results are available upon request

**Table 11:** Dietary energy consumption as alternative dependent variable

	(1)	(2)
Total trade openness (log)	433.7281 (2.64)***	
Cereals trade openness (log)		182.6741 (2.28)**
Non-cereals trade openness (log)		198.6428 (1.49)
GDP per capita (constant USD 2010)	0.0500 (3.38)***	0.0314 (1.75)*
Population growth	− 0.4880 (− 0.05)	8.9856 (0.69)
Rural population (%)	− 9.4442 (− 1.88)*	− 7.4147 (− 1.18)
Cereal yield (kg per ha)	0.0073 (0.44)	0.0216 (1.04)
Arable land (% of land area)	13.6555 (1.97)**	16.544 (2.15)**
Inflation based on Food Consumer Price Index	− 20.8333 (− 1.11)	30.1906 (1.12)
Inflation based on Cereals Production Price Index	− 107.1881 (− 1.20)	− 122.9282 (− 1.48)
Natural disasters	106.9815 (0.88)	− 36.2260 (− 0.30)
Conflicts	− 34.3469 (− 3.07)***	− 29.5408 (− 3.34)***
N of countries	71	71
N of observations	901	901
F	6.44***	7.49***
Anderson–Rubin Wald test (Chi squared *p* value)	0.0001	0.0001

Robust z statistic in parentheses

****p* < 0.01; ***p* < 0.05; **p* < 0.1

## Footnotes

1 Hunger was already mentioned in the predecessors of the SDGs, the Millennium Development Goals (Goal 1 “Eradicate extreme poverty and hunger”). However, it was only with the SDGs that food security has become an autonomous global goal for both developing and developed countries. The prevalence of undernourishment expresses the probability that a randomly selected individual from the population consumes an amount of calories that is insufficient to cover her/his energy requirement for an active and healthy life. The indicator is computed by comparing a probability distribution of habitual daily dietary energy consumption with a threshold level called the minimum dietary energy requirement. Both are based on the notion of an average individual in the reference population.

2 Target 2.1 is also measured by the prevalence of moderate or severe food insecurity in the population, based on the Food Insecurity Experience Scale. However, many data are still unavailable (SDG Indicator 2.1.2, UN, 2019b).

3 Also Mary (2019) divides the sample into net food-importing and exporting countries. However, the proportion between the two groups in his sample is not representative of the reality of developing countries, with an overrepresentation of the second group.

4 When our models are estimated considering food trade openness instead of cereals trade openness, results are similar but less statistically significant.

5 The option for this source of data follows Mary (2019) and is supported by the following advantages over alternative data sources: it provides figures about both trade and production values, thus ensuring some consistency when building the sectoral trade openness indexes; by employing mirror trade flows, it maximizes coverage and available observations through estimation of missing and unreported data.

6 Small countries presenting a population below half a million have been excluded from the sample.

7 Results of first-stage regressions are not reported but are available upon request.

8 This is calculated as (−0.1190/11.087)*100.

9 Unfortunately it was not possible to include a measure of income distribution among the regressors because of the scarce availability of data which would significantly reduce the size of our sample.

10 This is calculated as (0.0683/11.087)*100.

11 The lack of statistical significance may also be due to the fact that, in this specific case, the relevance of the instrument is not fully confirmed by the Anderson-Rubin Wald test.

12 The original intention was to drop the ten countries with the extremes values of undernourishment (i.e. the five with the highest values and the five with the lowest values). However, since the lowest value of undernourishment (2.5%) corresponds to 8 countries, we symmetrically dropped the 8 countries showing the highest values.

13 This is calculated as: (− 11.9001)*[ln(TTO_post_) − ln(TTO_pre_)]. The resulting change in the prevalence of undernourishment is then applied to the population of low- and middle-income countries as reported by the World Development Indicators (World Bank).

## Code availability

Information on the Stata code is available upon request.
